# Response to Booster Doses of Hepatitis B Vaccine among Young Adults Who Had Received Neonatal Vaccination

**DOI:** 10.1371/journal.pone.0107163

**Published:** 2014-09-08

**Authors:** Paul K. S. Chan, Karry L. K. Ngai, Terence T. Lao, Martin C. S. Wong, Theresa Cheung, Apple C. M. Yeung, Martin C. W. Chan, Scotty W. C. Luk

**Affiliations:** 1 Department of Microbiology, The Chinese University of Hong Kong, Hong Kong Special Administrative Region, China; 2 Department of Obstetrics and Gynaecology, The Chinese University of Hong Kong, Hong Kong Special Administrative Region, China; 3 School of Public Health and Primary Care, The Chinese University of Hong Kong, Hong Kong Special Administrative Region, China; 4 Faculty of Medicine; and University Health Service, The Chinese University of Hong Kong, Hong Kong Special Administrative Region, China; Saint Louis University, United States of America

## Abstract

**Background:**

Newborns who have received hepatitis B immunization in 1980s are now young adults joining healthcare disciplines. The need for booster, pre- and post-booster checks becomes a practical question.

**Aims:**

The aim of this study is to refine the HBV vaccination policy for newly admitted students in the future.

**Methods:**

A prospective study on medical and nursing school entrants to evaluate hepatitis B serostatus and the response to booster doses among young adults.

**Findings:**

Among 212 students, 17–23-year-old, born after adoption of neonatal immunization, 2 (0.9%) were HBsAg positive, 40 (18.9%) were anti-HBs positive. At 1 month after a single-dose booster for anti-HBs-negative students, 14.5% had anti-HBs <10 mIU/mL, 29.0% and 56.5% were 10–100 and >100 mIU/mL, respectively. The anti-HBs levels were significantly higher for females than males (mean [SD]: 431 [418] vs. 246 [339] mIU/mL, P = 0.047). At 2–4 month after the third booster dose, 97.1% had anti-HBs >100 mIU/mL and 2.9% had 10–100 mIU/mL.

**Conclusions:**

Pre-booster check is still worthwhile to identify carriers among newly recruited healthcare workers born after adoption of neonatal immunization. A 3-dose booster, rather than a single dose, is required for the majority to achieve an anti-HBs level >100 mIU/mL, as memory immunity has declined in a substantial proportion of individuals. Cost-effectiveness of post-booster check for anti-HBs is low and should be further evaluated based on contextual specific utilization of results.

## Introduction

More than 2 billion people worldwide have been infected with hepatitis B virus (HBV), and about 360 million are chronic carriers at risk of severe complications [1]. The adoption of universal neonatal immunization proves to be a successful strategy to reduce chronic hepatitis B infection and the consequent chronic liver diseases and primary hepatocellular carcinoma, especially in regions with historically high endemicity [2], [3]. Frontier countries have started such immunization policy as early as mid 1980s when safety and efficacy data on neonatal immunization were just available [4], [5]. As of 2006, about 27% of newborns worldwide received a birth dose of hepatitis B vaccine [6]. The World Health Organization emphasizes that all infants should receive hepatitis B vaccine soon after birth even in countries with intermediate or low endemicity [7]. While there is no evidence to support the need for booster following a complete course of neonatal immunization in general, special consideration for the at-risk groups may be worthwhile. Healthcare workers are regarded as a high-risk group for contracting hepatitis B infection because of exposure to blood and body fluids. Therefore vaccination is strongly recommended for non-immune healthcare workers regardless of endemicity in the area concerned [8], [9], [10].

In Hong Kong, neonatal immunization program was introduced in 1983. From 1983 to 1988, newborns of HBsAg-positive mothers received hepatitis B immunoglobulin and vaccination. From November 1988, this program was expanded to provide a full-course of HBV vaccine to newborns of HBsAg-negative mothers. Newborns who have received immunization in the 1980s are now young adults joining the healthcare disciplines or as entrants to medical and nursing schools. The need for booster vaccination for this neonatal vaccinated cohort, the number of doses, the value of pre- and post-vaccination check are practical questions.

## Methods

This study examined the pre-booster hepatitis B sero-status and antibody response to booster doses of vaccination among young adults. The study was conducted in a university where free pre-vaccination check for hepatitis B was routinely offered to all new entrants to healthcare-related programs. Students found to be negative for both hepatitis B surface antigen (HBsAg) and antibody to HBsAg (anti-HBs), defined as <10 mIU/mL, were offered a full course (3 doses) of vaccination free of charge.

Medical and nursing students enrolled in 2010 were invited to this study. The demographics, family, medical and hepatitis B vaccination history were recorded confidentially by a self-administered questionnaire. All students negative for both HBsAg and anti-HBs were offered 3 doses of 1 mL (20 mcg) ENGERIX-B (GlaxoSmithKline Biologicals, Belgium) at 0, 1, and 6 months. In addition to the routine pre-vaccination blood sample, students born after 1988 in Hong Kong (when universal neonatal HBV vaccination was launched) or had received neonatal vaccination elsewhere were invited on a voluntary basis to provide a blood sample at 1 month after the first dose of vaccination, immediately before receiving the second dose; and then between 2–4 months after the third dose. For the purpose of this study, students born after the adoption of neonatal immunization were tested for total antibody to hepatitis B core antigen (anti-HBcore). The hepatitis B seromarkers were detected by commercial kits (ARCHITECT HBsAg, Anti-HBs, AntiHBc II Kit, Abbott Diagnostic Division, Wiesbaden). The study was approved by the Joint Chinese University of Hong Kong-New Territories East Cluster Clinical Research Ethics Committee. We have obtained written consent from all subjects participated in this study. However, informed consent was not obtained from the next of kin, caretakers, or guardians on behalf of the 12 participants who were 17 years old as they were all university students and thus regarded as mature enough to understand the nature of this study. The consent procedure was approved by the ethics committee. All the other 402 participants were 18 years of age or older. Upon the completion of collection of the last blood sample, all study data were anonymized.

### Statistical Analysis

The positive rates for anti-HBs between groups were compared by Chi-squared test. The distribution of anti-HBs levels between genders was assessed by T-test. Correlation between anti-HBs levels after the first and third dose of booster vaccination was examined by paired samples T-test. Statistical analyses were performed by IBM SPSS v.18 (IBM, US). P-values of less than 0.05 were regarded as significant.

## Results

### Hepatitis B status of the whole cohort of new medical and nursing school entrants

**Figure 1:**
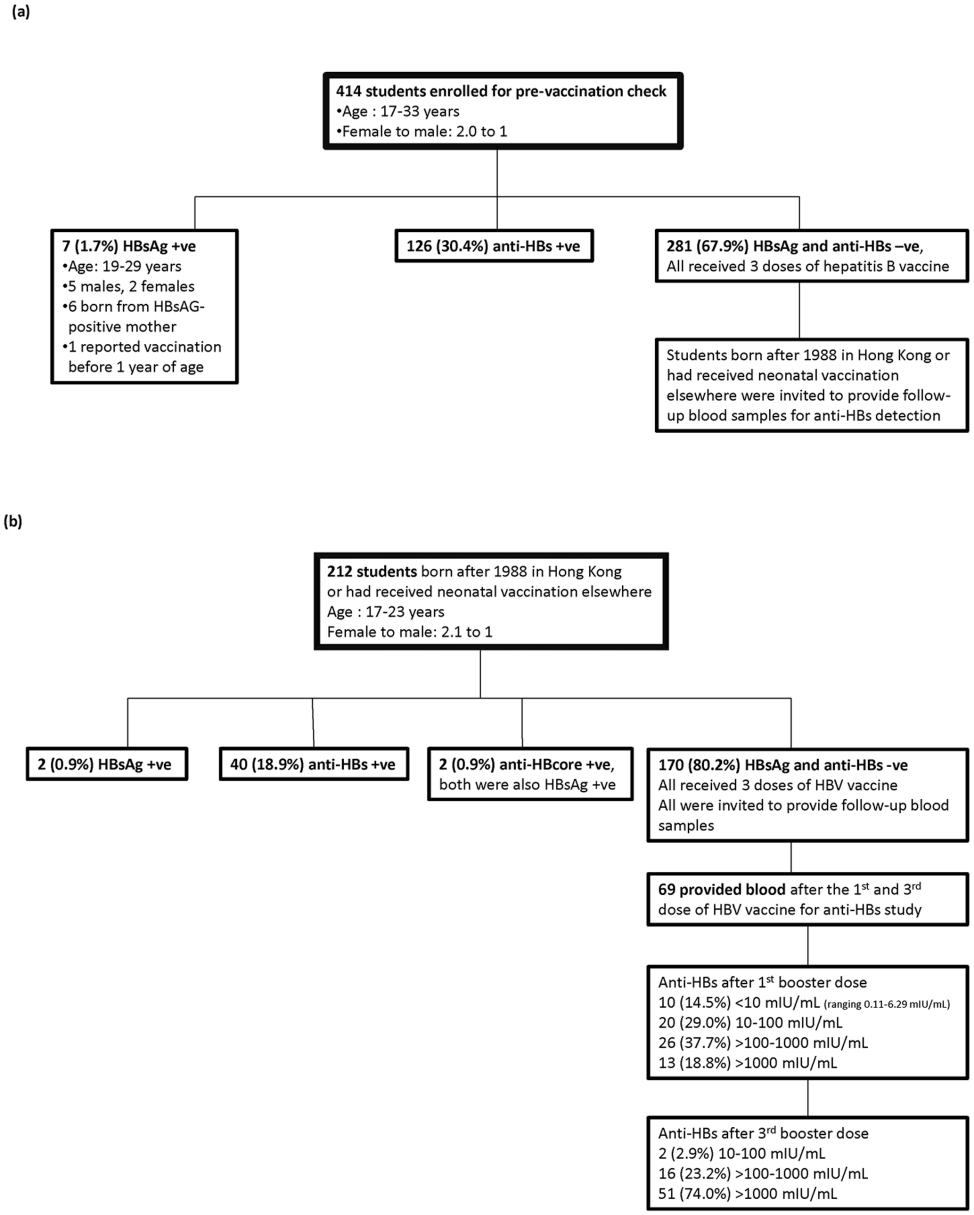
Summary of participant deposition, characteristics and hepatitis B status. (A) Whole cohort of new medical and nursing school entrants. (B) Cohort born after adoption of universal neonatal immunization.

Altogether, 414 students received pre-vaccination check (Figure 1a). Of these, 162 were medical and 252 were nursing students of age 17–33 (mean: 20) years, and with a female to male ratio of 2.0 to 1. Seven students (1.7%) aged 19–29 years, 2 females and 5 males, were found to be carriers (positive for HBsAg). Six of them were borne from HBsAg-positive mothers. Among the 4 carriers born in mainland China, 1 reported a history of vaccination in infancy. Of the 3 other carriers born in Hong Kong, 2 were born after the adoption of universal neonatal immunization, but the students were not certain about their vaccination history.

The overall positive rate for anti-HBs was 30.4% (126/414). Altogether, 67.9% (281/414) students negative for both HBsAg and anti-HBs, and all of them received 3 doses of HBV vaccine.

### Hepatitis B status of young adults born after adoption of universal neonatal immunization

Among the 272 students who had provided information on place of birth, 198 were born in Hong Kong after 1988 when universal neonatal immunization for hepatitis B was adopted, 14 were born elsewhere and with history of neonatal immunization. These 212 students formed the cohort for evaluation of hepatitis B serostatus among young adults born after adoption of neonatal immunization (Figure 1B). Their age ranged from 17 to 23 (mean: 19) years, with a female to male ratio of 2.1 to 1. Two were HBsAg positive (0.9%, 2/212) as described above. The positive rate for anti-HBs was 18.9% (40/212), with no significant difference between genders (P = 0.755 by Chi-squared test). Among the 40 students positive for anti-HBs, 22.5% (9/40) had levels of 10–100 mIU/mL, 37.5% (15/40) were >100–1000 mIU/mL, and 40.0% (16/40) were >1000 mIU/mL. Except the two carriers, all 210 students were negative for anti-HBcore.

### Response to single- and three-dose booster among young adults born after adoption of universal neonatal immunization

**Figure 2:**
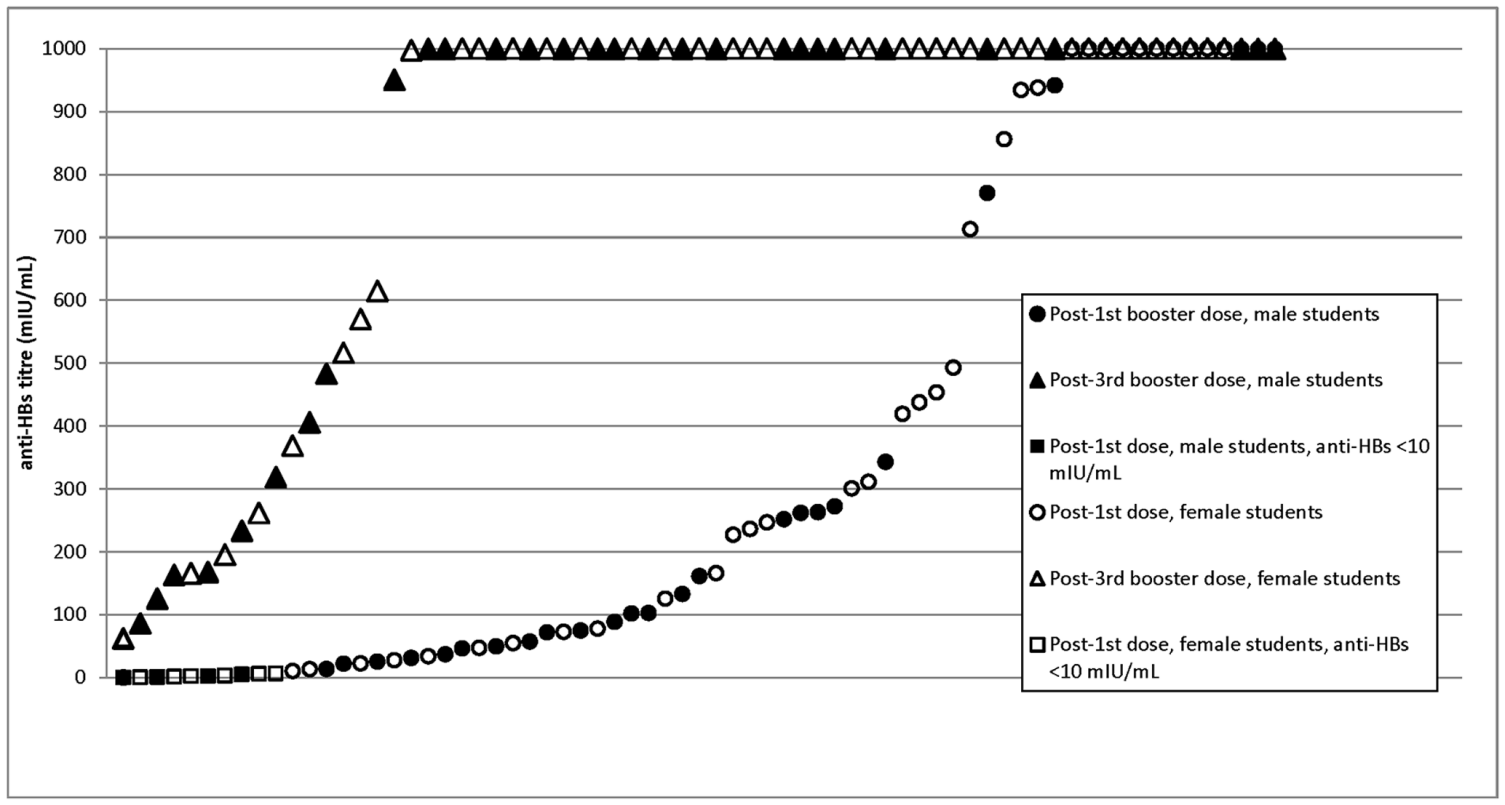
Levels of anti-hepatitis B surface antibody (anti-HBs) after booster vaccination for young adults. Each data point represents one student aged 17–23 years, born after the adoption of universal neonatal immunization for hepatitis B. All these 69 students were negative for HBsAg and anti-HBs before booster vaccination. Anti-HBs levels were determined one month after the first dose and 2–4 months after the third dose of booster vaccination with 20 mcg of ENERGIX-B (GlaxoSmithKline Biologicals, Belgium).

Altogether, 69 students born after adoption of universal neonatal immunization provided blood samples after the first and third dose of booster vaccination. At 1 month after the first booster dose, 10 (14.5%) students had anti-HBs levels of <10 mIU/mL (ranging from 0.11 to 6.29 mIU/mL), 20 (29.0%) had 10–100 mIU/mL, 26 (37.7%) had >100–1000 mIU/mL, and the remaining 13 (18.8%) were >1000 mIU/mL. The anti-HBs levels of female students were significantly higher than those of male students (geometric mean [standard deviation] = 431 [418] versus 246 [339] mIU/mL, P = 0.047 by T test without assuming normal distribution) (Figure 2).

After the completion of three doses of booster vaccination, 51 (74.0%) students had anti-HBs level >1000 mIU/mL, 16 (23.2%) were >100–1000 mIU/mL, and the remaining two students had 62 and 86 mIU/mL, respectively (Figure 2). A significant correlation between anti-HBs levels obtained after the first and third booster dose was observed (P<0.001 by paired samples T test without assuming normal distribution).

## Discussion

Hong Kong serves as an example of places with historically high endemicity of hepatitis B and universal neonatal immunization was started early in 1980s. The healthcare settings are facing a mixed prevalence of hepatitis B. On one hand, most patients are still within the age cohort of high carriage rate. For instance, as at 2009 the HBsAg positive rate of Hong Kong antenatal women was 8.2%, and that of tuberculosis patients was 9.5% [11]. On the other hand, the carriage rate among healthcare workers is decreasing and most new recruits have had neonatal vaccination that was launched in 1988. The necessity and cost-effectiveness of continuing screening for every new recruit need to be evaluated.

We found that a small proportion (0.9%) of new medical and nursing students born after adoption of neonatal immunization were positive for HBsAg. Identifying these students is necessary not only for medical follow-up, but it also has implications on their choice of specialty in the future. At present, unlike countries with low endemicity [12], Hong Kong does not impose restrictions on clinical practice based on the hepatitis B status of healthcare workers. However, this may change in the future when the proportion of carriers become small enough to make such policy feasible.

We found that the overall positive rate for anti-HBs among these young university entrants was 30.4%, which was higher than the cohort born after adoption of universal neonatal HBV immunization (18.9%). While our study cannot provide a define reason for this observation, we recorded that 11% of the non-neonatal vaccinated cohort had received HBV vaccine in recent years, and majority (63%) of them remained positive for anti-HBs.

In line with some previous reports [13], [14], we found that only a small proportion (18.9%) of young adults who had received neonatal immunization remained positive for anti-HBs. The current view of World Health Organization is that these persons still retain memory immunity, and booster dose is not necessary in routine immunization program [7]. Our observation is in line with this, since despite a relatively low anti-HBs positive rate among the neonatal vaccinated cohort, anti-HBcore was negative in all except two carriers. Nevertheless, for healthcare workers who are at continuous risk of exposure to hepatitis B infection, a more conservative approach has been adopted in some countries, where a regular booster is recommended [10].

Our data showed that a single-dose booster for anti-HBs-negative young adults who had received neonatal vaccination might not be sufficient. We found that after a single-dose booster, only 56.5% developed an anti-HBs level of >100 mIU/mL that is regarded a preferable post-immunization level for healthcare workers, though 10 mIU/mL is generally accepted as protective [10]. Furthermore, 14.5% of the students had anti-HBs levels of <10 mIU/mL after a single dose booster suggesting lost of memory immunity. Previous studies on teenagers who had received infantile vaccination also indicated a single-dose booster could not reliably induce anti-HBs response suggesting that the memory immunity, at least the humoral part, wanes with time [13], [14], [15].

In line with a previous study in Taiwan [16], we found that the anti-HBs response rate to 3 doses of booster vaccine was 100%. This raises a question on the cost-effectiveness of post-booster check. Of note, a small proportion (2.9%) of responders had anti-HBs below 100 mIU/mL. The recommendation for this group varies among authorities. The more conservative one recommends a fourth dose [10]. If this approach is adopted, there will be one additional justification to perform post-booster check, on top of providing a proof of response for managing sharps injury incidents in the future. The actual savings from reducing unnecessary administration of hepatitis B immunoglobulin need to be assessed based on contextual specific data.

A policy of hepatitis B vaccination for healthcare workers including medical and nursing students has been established in most countries. In view of the fact that coming cohorts have already received neonatal vaccination, such policy should be revisited. The results of this study suggest that a pre-vaccination check is still worthwhile to pick up the small number of carriers that exist because of various reasons including the rare failure in protection or uptake, or immigrants from countries without universal immunization. The anti-HBs titer necessary to protect people in a high risk occupation (health care workers) in an area of high endemicity, such as Hong Kong, is not known with certainty, but titers >100 mIU/mL is a reasonable recommendation, and our data show that a 3-dose booster series is necessary to achieve this in the majority of individuals who are negative for anti-HBs. In addition, we recommend a more conservative approach to offer a single-dose booster to those positive (≥10 mIU/mL) for anti-HBs or at least for those with levels of 10–100 mIU/mL, especially in settings where regular booster is not reinforced. The cost-effectiveness of post-vaccination check may be low. However, this cost can be justified if documentation of response to vaccination will save the cost and potential risk of unnecessary administration of hepatitis B immunoglobulin.

Re-enforcing vaccination for adults is difficult. Entrance to medical and nursing schools is a good opportunity to implement vaccination policy for hepatitis B as well as other infections.
